# Post-COVID postural orthostatic tachycardia syndrome (POTS): a new phenomenon

**DOI:** 10.3389/fneur.2024.1297964

**Published:** 2024-03-22

**Authors:** Christopher Cantrell, Conor Reid, Claudia S. Walker, Samantha J. Stallkamp Tidd, Ryan Zhang, Robert Wilson

**Affiliations:** ^1^Cleveland Clinic Lerner College of Medicine, Cleveland, OH, United States; ^2^Keck School of Medicine, University of Southern California, Los Angeles, CA, United States; ^3^Department of Neuromuscular Medicine, Cleveland Clinic Foundation, Cleveland, OH, United States

**Keywords:** POTS, autonomic, neurology, orthostatic, COVID, post-COVID, long-hauler

## Abstract

**Background:**

The impact of COVID-19 has been far-reaching, and the field of neurology is no exception. Due to the long-hauler effect, a variety of chronic health consequences have occurred for some post-COVID patients. A subset of these long-hauler patients experienced symptoms of autonomic dysfunction and tested positive for postural orthostatic tachycardia syndrome (POTS) via autonomic testing.

**Methods:**

We conducted a chart review of a convenience sample from patients seen by neurologists at our tertiary care center for suspicion of post-COVID POTS. Patients included in our study had clearly defined POTS based on clinical criteria and positive tilt table test, were 81.25% female, and had an average age of approximately 36. Out of 16 patients, 12 had a confirmed positive COVID test result, with the remaining 4 having strong clinical suspicion for COVID infection. Our analysis examined the most bothersome 3 symptoms affecting each patient per the neurologist’s note at their initial visit for post-COVID POTS, clinical presentation, comorbidities, neurological exam findings, autonomic testing results, and COMPASS-31 autonomic questionnaire and PROMIS fatigue survey results.

**Results:**

Palpitations (68.75%) and fatigue (62.5%) were the most common of the impactful symptoms reported by patients in their initial Cleveland Clinic neurology visit. The most frequent comorbidities in our sample were chronic migraines (37.5%), irritable bowel syndrome (IBS) (18.75%), and Raynaud’s (18.75%). Neurological exam findings and autonomic testing results other than tilt table yielded variable findings without clear trends. Survey results showed substantial autonomic symptom burden (COMPASS-31 autonomic questionnaire average score 44.45) and high levels of fatigue (PROMIS fatigue survey average score 64.64) in post-COVID POTS patients.

**Conclusion:**

Our sample of post-COVID POTS patients are similar to the diagnosed POTS general population including in comorbidities and autonomic testing. Fatigue was identified by patients as a common and debilitating symptom. We hope that our study will be an early step toward further investigation of post-COVID POTS with focus on the trends identified in this chart review.

## 1 Introduction

Postural orthostatic tachycardia syndrome (POTS) is a systemic condition of autonomic dysfunction that leads to orthostatic intolerance with upright posture associated with rapid heartbeat increase in the absence of orthostatic hypotension (1, 2). Theories of POTS pathophysiology include heightened sensitivity of cardiac beta-adrenoreceptors, cardiovascular deconditioning, peripheral neuropathy, autoimmunity and immune dysregulation (3, 4). In the United States, POTS has predominantly been diagnosed in white females between 15 and 45 years of age (4, 5). Though it is approximated that around 1–3 million people in the U.S. are affected by POTS, the demographics and potential disparities in diagnosis between patient populations has yet to be thoroughly investigated (1, 5). Immunological stressors, such as viral infections, vaccination, trauma, surgery, pregnancy, or psychosocial stress, have been associated with disease onset (4). The most common symptoms reported by POTS patients are dizziness and palpitation on standing with rapid heartbeat and weakness (4). Additional symptoms include headache, brain fog, dyspnea, physical decondition, GI symptoms, and musculoskeletal pain (4).

Of POTS patients responding to a large-scale online survey, 63% felt they had experienced POTS-like symptoms for most of their lives (5). In that survey study, 44% experienced worsening of POTS symptoms, 10% claimed no significant change in symptom burden since disease onset, and 42% reported symptom improvement over time (29% of those patients claimed medications were most responsible for their improvement) (5). Diagnostic criteria have been developed characterizing the clinical presentation of POTS (1). The title table test typically serves as the first-line for diagnostic testing of one’s autonomic function, while QSART (Quantitative Sudomotor Axon Reflex Test), Valsalva, and catecholamine level testing serve as additional testing options (2, 4). Common comorbidities include Sjogren’s disease, celiac disease, Hashimoto’s, rheumatoid arthritis, Ehlers-Danlos, Chiari malformation, Raynaud’s, migraine, fibromyalgia, IBS, and IBD (1, 6). Generalized symptoms, comorbidities, and seeing many different doctors during the workup process often result in missed diagnoses and delayed patient care for this debilitating condition (5).

As COVID swept through our population, the long-hauler phenomenon gained increasing prevalence (7). A survey in post-COVID patients, the vast majority of whom had mild symptoms, showed that nearly a third of the participants had at least one persistent symptom after resolution of their COVID infection. Fatigue was a commonly reported long-COVID symptom, and about 1 in 12 patients experienced decreased ability to perform an activity of daily living such as household chores (8). Autonomic dysfunction is a common consequence suffered by COVID long-haulers, manifesting in symptoms including headache, fatigue, orthostatic intolerance, tachycardia/palpitations, temperature intolerance, and more, reflecting the growing incidence of POTS pathophysiology in these patients (7).

As a debilitating manifestation of post-viral autonomic disease, post-COVID POTS is an important area of research to learn about these patients, their condition, and how to treat them. Here, we characterize a sample of patients who presented to our tertiary care center after COVID-19 with autonomic symptoms that met diagnostic criteria for POTS. We hope to offer a novel glimpse into the clinical presentation of post-COVID POTS patients to improve diagnosis and foster further research into the prognosis and treatment of those with this condition.

## 2 Methods

### 2.1 Sample

The patients selected for our sample initially presented to the neurology department at our institution for suspicion of autonomic dysfunction between September 2020 and July 2021, and have since had confirmed diagnoses of POTS. This convenience sample was selected to provide a cohort large enough to discover potential trends given the data readily accessible to our neurology research team via the EPIC electronic medical records. We selected our sample by identifying patients who closely fit the diagnostic criteria for POTS, including clinical presentation and diagnostic testing. Symptoms associated with POTS include palpitations, orthostatic intolerance, syncope, headache, chest pain, GI symptoms, anxiety, and shortness of breath. For symptom-based analysis, only the three most impactful self-reported symptoms were recorded for each patient. These symptoms were determined based on the history in the neurologist’s note from the initial visit for suspected post-COVID POTS. The language used in the note assisted in identifying the most substantial symptoms, with priority given to symptoms listed earlier in the history to help differentiate if needed. Comorbidities were identified through chart review based on past medical history prior to illness with COVID-19: chronic migraine, celiac disease, Hashimoto’s thyroiditis, rheumatoid arthritis, Chiari malformation, Sjogren’s disease, Raynaud’s, fibromyalgia, Ehlers-Danlos syndrome, Crohn’s disease, IBS, and IBD.

### 2.2 Reviewed testing

The tilt table test is the current standard for POTS diagnostic testing. First, an average heart rate is obtained while the patient is supine for 5 min. Next, the patient is tilted upward by 70 degrees and their average heart rate is measured each minute over a period of 10 min (9). The threshold for a positive test result involves calculating the difference between the average supine heart rate and the maximum heart rate measurement over the 10 min during which the patient is tilted. Tilt table is diagnostic for POTS if within this 10-min time, the patient has a sustained heart rate increase of at least 30 beats per minute in the absence of orthostatic hypotension or another condition that may explain the sinus tachycardia. In addition, the patient must have frequent orthostatic symptoms upon standing with resolution upon sitting that have persisted for at least 3 months (1). All 16 patients in this study fit the diagnostic criteria for POTS including positive tilt table testing. Detailed results of the tilt table test are provided in Table 1.

**Table 1:** Tilt table detailed results (*n* = 16).

Mean max heart rate (supine)	59 bpm
Mean max heart rate (tilt)	93 bpm
Mean max heart rate difference	34 bpm
Mean SBP (supine)	114 mmHg
Mean DBP (supine)	70 mmHg
Mean max SBP (tilt)	135 mmHg
Mean min SBP (tilt)	124 mmHg
Mean max DBP (tilt)	99 mmHg
Mean min DBP (tilt)	86 mmHg

QSART (Quantitative Sudomotor Axon Reflex Test) is an autonomic test evaluating sudomotor function to assess for small autonomic nerve fiber damage. This test is performed by electrically stimulating the skin to induce acetylcholine release, which ordinarily induces sweating. An abnormal response is detected if the sweat production measured during the QSART is below a normal threshold that accounts for age and sex. An abnormal QSART result suggests the patient is experiencing post-ganglionic sympathetic fiber dysfunction (10). The results for each sample taken from the patient are compared to reference values. For sweat latency, the reference values in minutes and seconds are: forearm 1:30–3:30, proximal leg 0:50–2:20, distal leg 0:50–2:20, and proximal foot 1:20–2:30. For sweat output, the normal values in mL/cm^2^ are forearm >0.08, proximal leg >0.19, distal leg >0.14, and proximal foot >0.07. Detailed mean results of the QSART test for our study sample are displayed in Table 2.

**Table 2:** Quantitative Sudomotor Axon Reflex Test (QSART) detailed results.

QSART measurement	Mean (*n* = 8)
Forearm sweat latency	1 min 45 s
Forearm sweat output	0.3925 mL/cm^2^
Proximal leg sweat latency	1 min 27 s
Proximal leg sweat output	0.43375 mL/cm^2^
Distal leg sweat latency	1 min 36 s
Distal leg sweat output	0.42125 mL/cm^2^
Proximal leg sweat latency	2 min 46 s
Proximal leg sweat output	0.21375 mL/cm^2^

Skin punch biopsy (SPB) is performed to search for small fiber neuropathy in the patient’s skin. In a subset of our cohort, two skin punch biopsies 3 mm in size were taken from the patient’s distal leg and distal thigh. Our clinic’s laboratory then assessed epidermal nerve fiber density in each sample to look for reduced density suggestive of small fiber neuropathy (11). A sample was deemed to have reduced epidermal nerve fiber density when below the 5th percentile (distal leg <5 fibers/mm or distal thigh <7 fibers/mm).

Deep Breathing autonomic testing involves the subject laying supine and taking 6 deep breaths over 1 min while measuring the heart rate. This specifically tests function of the vagal nerve. Respiratory sinus arrhythmia (RSA) is determined by calculating the difference in heart rate between the end of expiration and the end of inspiration. Normal values for RSA for the deep breathing test are stratified by age (11, 12). Detailed results of the Deep Breathing test are shown in Table 3.

**Table 3:** Valsalva and deep breathing test detailed results.

Valsalva test	*n* = 10
Valsalva ratio	Mean = 2.266
Blood pressure response phase II	Normal 100%
Blood pressure response phase IV	Normal 100%
Deep breathing test	*n* =10
Mean heart rate range	21.372
Expiratory/inspiratory range	1.43

The Valsalva test requires the patient to perform the Valsalva maneuver, which involves exhaling against a closed airway to raise intrathoracic pressure. The reduced preload to the heart can activate the baroreceptor reflex. The output of patient Valsalva testing is compared to established reference values as described in Novak 2011 to determine whether the results are normal or abnormal (12). The reference values vary based on patient age. Table 3 contains detailed results of the Valsalva test for our study sample.

To characterize the clinical presentation of our patient sample, we conducted chart review beyond symptoms and autonomic testing to include demographic characteristics, neurological exam findings, common comorbidities, and survey results as described below. The following neurological exam findings were reviewed: pinprick, temperature perception, length-dependent neuropathy, light touch sensation, vibratory sense, reflexes (hyporeflexia and hyperreflexia), proprioception, and Romberg test.

### 2.3 Reviewed questionaries

The COMPASS-31 questionnaire measures patient-reported outcomes related to autonomic symptoms. This questionnaire has 31 questions and is scored out of 100 total points. A higher score indicates more severe autonomic symptoms. The 31 questions investigate the presence, frequency, and severity of symptoms divided between 6 domains: orthostatic intolerance, vasomotor, secretomotor, gastrointestinal, bladder, and pupillomotor (13). The domain of orthostatic intolerance consists of dizziness or feeling faint upon standing. Vasomotor questions on the COMPASS-31 survey investigate skin color changes. The secretomotor domain regards sweating changes, dry eyes, and dry mouth. Gastrointestinal symptoms assessed are bloating, vomiting, colicky abdominal pain, diarrhea, and constipation. The bladder domain surveys for incontinence, difficulty passing urine, and incomplete emptying. Lastly, the pupillomotor section involves light sensitivity and trouble focusing the eyes.

The PROMIS 10a fatigue survey provides a score to assess the symptom burden of fatigue experienced by patients. T-scores are calculated based on the survey results, with a T-score of 50 correlating with the population mean and a standard deviation of 10. A higher score indicates greater fatigue (14, 15).

## 3 Results

The convenience sample of post-COVID POTS patients was comprised of 16 patients with a mean age of 36.06 (range: 18–52). Our sample of 16 patients was primarily white (93.75%) and female (81.25%). 75% of the patient sample had confirmed positive COVID by PCR testing logged in the electronic medical record, while the remainder of patients were strongly suspected of having COVID due to timing of infection, known exposures, and/or presenting symptoms. The mean BMI in the group was 24.53 (range: 16.29–33.37). Mild severity of COVID infection (as designated in a physician note within the electronic medical record) was seen in 93.75% of those in our study. Anxiety (75%) and depression (62.5%) were common pre-COVID diagnoses in our post-COVID POTS patients (Table 4).

**Table 4:** Characteristic of post-COVID POTS patient sample (*n* = 16).

Characteristics	Value
Total number of patients	16
Age at presentation: average (st dev)	36.06 (9.24)
BMI: average (st dev)	24.53 (5.33)
White (%)	93.75%
Female (%)	81.25%
Confirmed COVID positive (%)	75%
Mild COVID severity (%)	93.75%
Diabetes (%)	0%
Anxiety (%)	75%
Depression (%)	62.5%

The symptoms that most prominently affected patients in our study based on the neurologist’s note from the initial visit for suspected post-COVID POTS varied between our 16 patients. The most common high-impact symptoms were palpitations (68.75%), fatigue (62.5%), and dyspnea (37.5%). The next most prevalent symptoms were headache (25%) and syncope/presyncope (25%). Cognitive changes (18.75%) and paresthesia (18.75%) were less common as most bothersome symptoms, as was dizziness (12.5%). Weakness and light sensitivity (6.25%) were rare. Among the three most substantial symptoms listed in the neurologist’s initial notes for our patients were none of the following: tremors, heat intolerance, postural lightheadedness, anxiety, joint pain, and sensory overload. These results are detailed in Table 5.

**Table 5:** Three most impactful symptoms in post-COVID POTS patients based on physician note (*n* = 16).

Symptom	% of patients
Palpitations	68.75%
Fatigue	62.5%
Dyspnea	37.5%
Headache	25%
Syncope/presyncope	25%
Cognitive changes	18.75%
Paresthesia	18.75%
Dizziness	12.5%
GI symptoms	6.25%
Chest pain	6.25%
Weakness	6.25%
Light sensitivity	6.25%
Tremors	0%
Heat intolerance	0%
Postural lightheadedness	0%
Anxiety	0%
Joint pain	0%
Sensory overload	0%

The two comorbidities in our 16-patient sample most frequently seen in our study were chronic migraine (37.5%) and IBS (18.75%). Raynaud’s phenomenon (18.75%) was also common in our study sample. Ehlers-Danlos (12.5%), Sjogren’s (12.5%), and Hashimoto’s (12.5%) were present in our patients as well. Celiac disease (6.25%) and fibromyalgia (6.25%) were less common among this cohort. Not diagnosed in our group of 16 patients were Chiari malformation, rheumatoid arthritis, Crohn’s disease, and colitis (Table 6).

**Table 6:** Comorbidity percentage among post-COVID POTS patients (*n* = 16).

Comorbidity	% of patients
Chronic migraine	37.5%
IBS	18.75%
Raynaud’s	18.75%
Ehlers-Danlos	12.5%
Sjogren’s	12.5%
Hashimoto’s	12.5%
Celiac	6.25%
Fibromyalgia	6.25%
Chiari malformation	0%
Rheumatoid arthritis	0%
Chron’s	0%
Colitis	0%

Neurological exam findings from the initial post-COVID POTS visit to our neurology clinic were variable and completed in a subset of patients in our study (Table 7). Of the patients who underwent neurological examination, hyperreflexia was present in 16.67% (*n* = 12) and hyporeflexia was noted for 25% (*n* = 12) with nearly all abnormal reflexes at either 1+ or 3+. Length-dependent neuropathy and diminished light touch sensation were found in 27.27% of 11 patients. Less common was loss of pinprick sensation (25%, *n* = 8), in addition to decreased temperature perception and vibratory sense (10%, *n* = 10). Proprioception deficits (*n* = 7) and positive Romberg testing (*n* = 12) were not found in our sample of POTS patients.

**Table 7:** Neurological exam findings in post-COVID POTS patients.

Exam component	Positive findings (%)
Hyperreflexia (*n* = 12)	16.67%
Hyporeflexia (*n* = 12)	25%
Length-dependent neuropathy (*n* = 11)	27.27%
Light touch (*n* = 11)	27.27%
Pinprick (*n* = 8)	25%
Temperature perception (*n* = 10)	10%
Vibratory sense (*n* = 10)	10%
Proprioception (*n* = 7)	0%
Romberg test (*n* = 12)	0%

Thirty-nine patients who had visited our institution’s neurology clinic for suspected post-COVID POTS were screened for inclusion in the study. Of those 39 patients, only those with a positive tilt table test (*n* = 16) were included per the study inclusion criteria. The remaining autonomic tests were completed in varying subsets of the study group. QSART testing was positive in 62.5% of those tested (*n* = 8), and skin punch biopsy showed neuropathic findings in 40% (*n* = 5). Deep breathing testing showed 10% abnormal results (*n* = 10) with no significant discrepancies in Valsalva testing (*n* = 10). The post-COVID POTS patients who took the COMPASS-31 autonomic questionnaire showed an average score of 44 (*n* = 7). Of the patients who took the PROMIS fatigue survey, the t-score averaged to 64.64 (*n* = 11), which is higher than the first standard deviation of 10 above the population mean set at 50 (Table 8).

**Table 8:** Autonomic testing and survey results for post-COVID POTS patients.

Autonomic test	Positive result ratio (#/n) (%)
Tilt table	16/16 (100%)
QSART	5/8 (62.5%)
Skin punch biopsy	2/5 (40%)
Deep breathing	1/10 (10%)
Valsalva	0/10 (0%)
Survey	Average score
COMPASS-31 autonomic questionnaire (*n* = 7)	44.45
PROMIS fatigue survey (*n* = 11)	64.64

## 4 Discussion

The goal of this study was to identify the leading clinical and symptom presentation of post-COVID POTS patients. As a condition with highly variable clinical presentations, we targeted analysis of post-COVID POTS symptoms to the top three most impactful symptoms as described on the physician note from the patient’s initial visit to our neurology department. By focusing on the most impactful symptoms identified in the patient visit, our hope was to identify what has the strongest effect on this patient population, which someday may have important implications for treatment and optimizing patient quality of life.

Other initial investigations of post-COVID POTS have suggested interesting trends. Most post-COVID patients have autonomic symptoms detectable by testing (16). One study reported that while orthostatic intolerance was highly prevalent in post-COVID patients, testing failed to show the expected hemodynamic changes for this symptom (16). It is clear that severe fatigue is one of the highest impact factors in post-COVID POTS and limits patients’ ability to fulfill independent activities of daily living and occupational function (17, 18). The impact of long-COVID has also been evaluated, such as lingering autonomic symptoms being found in 85% of post-COVID patients with 60% being unable to return to work at 6–8 months after resolution of their infection (19). This case series only had 20 patients, but the symptom burden is clear even with a small sample size (19). Altogether, between our findings and those of other studies, this suggests that autonomic testing is indicated and important for post-COVID patients with symptoms of autonomic nervous system dysregulation to allow for early intervention if POTS testing returns as positive.

Comorbidity analysis yielded interesting trends in our limited sample of post-COVID POTS patients. In particular, chronic migraine and IBS were among the most selected comorbidities for prevalence in our patient sample prior to their infection with COVID-19. Migraines may induce autonomic symptoms during the episodic pain attacks (20). Irritable bowel syndrome is a disorder of visceral hypersensitivity (21). Further investigation is necessary to determine any conclusions regarding the trends noticed in our study sample.

Autonomic testing often shows variable results among POTS patients, and this tendency proved to be true for our cohort of post-COVID POTS patients as well (6). The QSART and skin punch biopsy were positive in approximately half of those tested in our study sample, but these tests are not expected to be positive in every patient. Our interpretation of these limited results is that they are consistent with the POTS patient population in general. Further studies with large sample sizes would be required to determine if there is a difference in autonomic testing between post-COVID POTS patients and the general POTS population.

Neurological physical exam findings can be helpful in narrowing down differential diagnoses, but in our sample, the results were widely variable. Having heightened or suppressed reflexes, reduced pinprick sensation, or length-dependent neuropathy on exam are not features that could individually identify post-COVID POTS, and no neurological exam findings were found in more than half of our sample. Further characterization of this new patient population may reveal more trends, but based on our convenience sample, the exam findings support the heterogeneous presentation of post-COVID POTS.

The COMPASS-31 autonomic questionnaire score average indicates a significant autonomic symptom burden in this group of patients. This is to be expected in POTS patients, who will suffer from dysautonomia regardless of whether the inciting factor is COVID or not. Future work should investigate whether the COMPASS-31 survey score changes in post-COVID patients over their recovery and if this measure holds prognostic value.

The PROMIS fatigue survey showed a significant impact of fatigue on the subset of patients who took the questionnaire. The average score was more than one standard deviation above the population mean. This supports the narrative of fatigue as a prominent symptom of post-COVID POTS. Even with 11 patients in our sample taking the PROMIS fatigue survey, there is a clear impact of fatigue on these patients’ daily life.

A key takeaway from our study is that our post-COVID POTS study sample reflects the general POTS patient population. Similar to our study sample, patients diagnosed with POTS seen by neurologists at our institution have shown high percentage of being female (>80%) and white (>87%) (6). In a general POTS study performed at our institution, over half of POTS patients had comorbid migraines, along with frequent autoimmune comorbidities such as Sjogren’s, Hashimoto’s, and celiac disease (6). Also seen in the POTS patients of that study was fibromyalgia (around 10–33% by different subgroups) in addition to about 33% abnormal QSART testing and 24% abnormal skin punch biopsy results (6). This is the most direct comparison we can perform between general POTS patients and post-COVID POTS patients seen at our institution, and at this time we are unable to identify a notable differentiating factor between post-COVID POTS and POTS in general. By ensuring that each patient included in this study had a positive tilt table test, we limited our sample size to 16. However, this choice was intended to increase the validity of the trends we have reported. The top 3 symptoms approach to assessing post-COVID POTS has an inherently subjective component, but we wanted to focus on the impact of the disease and what patients felt most hindered their daily functioning. It is important to note that our analysis of testing results was limited by not all patients having each autonomic test, survey, or neurological exam component. The reason for these inconsistencies was based on the available information in the medical record and differences in patient follow-up with the usual progression of POTS work-up and care. In addition, only 12 out of 16 patients in our study were confirmed COVID positive. The remaining four patients’ COVID diagnosis was based on strong clinical suspicion based on symptoms and known sick contacts. These four patients likely were not tested for COVID due to isolation and the mild nature of their symptoms. However, their clinical diagnosis and the timing of POTS symptomatic onset warrant their inclusion in this study. One core element of the variable testing described above was the impact of the COVID-19 pandemic on non-essential diagnostic testing. With the danger of increased patient volume and the travel required for many of our patients, in some situations it was safer to avoid this testing at the time.

This study is an early step meant to inspire future investigation. Although limited, our study demonstrates that post-COVID POTS is heterogenous in its presentation but carries a significant disease burden. Future work should focus on following post-COVID POTS over time and identifying prognostic factors in addition to high quality treatments.

As COVID continues to infect more individuals in the U.S., more people become vulnerable to post-viral syndromes, including the potential development of POTS pathophysiology. In our study, fatigue, palpitations, and dyspnea were identified as the most prominently debilitating symptoms. Chronic migraine and IBS were the most common comorbidities in our patient sample. Neurological exam findings and autonomic testing results were non-specific and variable. Average patient-reported outcomes were notable on the COMPASS-31 questionnaire and PROMIS fatigue survey. Overall, the findings from this chart review study point toward a variable clinical presentation of post-COVID POTS. It is essential that further investigation is conducted to gain a better understanding of post-COVID POTS patients and their clinical presentation. Most important is that we listen to their stories and let them feel heard in their journey toward finding their new normal.

## Data availability statement

The raw data supporting the conclusions of this article will be made available by the authors, without undue reservation.

## Ethics statement

The studies involving humans were approved by the Cleveland Clinic Institutional Review Board. The studies were conducted in accordance with the local legislation and institutional requirements. Written informed consent for participation was not required from the participants or the participants’ legal guardians/next of kin in accordance with the national legislation and institutional requirements.

## Author contributions

CC: Conceptualization, Investigation, Methodology, Visualization, Writing – original draft, Writing – review & editing. CR: Investigation, Writing – review & editing. CW: Investigation, Writing – review & editing. SS: Writing – review & editing. RZ: Methodology, Writing – review & editing. RW: Conceptualization, Funding acquisition, Project administration, Supervision, Writing – review & editing.

## References

[1] Vernino S Bourne KM Stiles LE Grubb BP Fedorowski A Stewart JM. Postural orthostatic tachycardia syndrome (POTS): state of the science and clinical care from a 2019 National Institutes of Health expert consensus meeting – part 1. Auton Neurosci Basic Clin. (2021) 235:102828. doi: 10.1016/j.autneu.2021.102828, PMID: 34144933 PMC8455420

[2] Olshansky B Cannom D Fedorowski A Stewart J Gibbons C Sutton R. Postural orthostatic tachycardia syndrome (POTS): a critical assessment. Prog Cardiovasc Dis. (2020) 63:263–70. doi: 10.1016/j.pcad.2020.03.010, PMID: 32222376 PMC9012474

[3] Low PA Sandroni P. Chapter 106 - postural tachycardia syndrome (POTS) In: Robertson D Biaggioni I Burnstock G Low PA Paton JFR, editors. Primer on the autonomic nervous system. 3rd ed. San Diego: Academic Press (2012). 517–9.

[4] Fedorowski A. Postural orthostatic tachycardia syndrome: clinical presentation, aetiology and management. J Intern Med. (2019) 285:352–66. doi: 10.1111/joim.12852, PMID: 30372565

[5] Shaw BH Stiles LE Bourne K Green EA Shibao CA Okamoto LE. The face of postural tachycardia syndrome – insights from a large cross-sectional online community-based survey. J Intern Med. (2019) 286:438–48. doi: 10.1111/joim.12895, PMID: 30861229 PMC6790699

[6] Zhang R Mayuga K Shields R Cantrell C Wilson R. Skin biopsy and quantitative Sudomotor axon reflex testing in patients with postural orthostatic tachycardia syndrome. Cureus. (2022) 14:e31021. doi: 10.7759/cureus.31021 36349067 PMC9629858

[7] Larsen NW Stiles LE Miglis MG. Preparing for the long-haul: autonomic complications of COVID-19. Auton Neurosci. (2021) 235:102841. doi: 10.1016/j.autneu.2021.102841 34265539 PMC8254396

[8] Logue JK Franko NM McCulloch DJ McDonald D Magedson A Wolf CR. Sequelae in adults at 6 months after COVID-19 infection. JAMA Netw Open. (2021) 4:e210830. doi: 10.1001/jamanetworkopen.2021.0830, PMID: 33606031 PMC7896197

[9] Thijs RD Brignole M Falup-Pecurariu C Fanciulli A Freeman R Guaraldi P. Recommendations for tilt table testing and other provocative cardiovascular autonomic tests in conditions that may cause transient loss of consciousness. Clin Auton Res. (2021) 31:369–84. doi: 10.1007/s10286-020-00738-6, PMID: 33740206 PMC8184725

[10] Illigens BMW Gibbons CH. Sweat testing to evaluate autonomic function. Clin Auton Res Off J Clin Auton Res Soc. (2009) 19:79–87. doi: 10.1007/s10286-008-0506-8, PMID: 18989618 PMC3046462

[11] Gibbons CH Bonyhay I Benson A Wang N Freeman R. Structural and functional small Fiber abnormalities in the neuropathic postural tachycardia syndrome. PLoS One. (2013) 8:e84716. doi: 10.1371/journal.pone.0084716, PMID: 24386408 PMC3874039

[12] Novak P. Quantitative autonomic testing. J Vis Exp JoVE. (2011) 53:2502. doi: 10.3791/2502 PMC3196175 21788940

[13] Sletten DM Suarez GA Low PA Mandrekar J Singer W. COMPASS 31: a refined and abbreviated composite autonomic symptom score. Mayo Clin Proc. (2012) 87:1196–201. doi: 10.1016/j.mayocp.2012.10.013, PMID: 23218087 PMC3541923

[14] Christodoulou C Junghaenel DU DeWalt DA Rothrock N Stone AA. Cognitive interviewing in the evaluation of fatigue items: results from the patient-reported outcomes measurement information system (PROMIS). Qual Life Res Int J Qual Life Asp Treat Care Rehabil. (2008) 17:1239–46. doi: 10.1007/s11136-008-9402-x, PMID: 18850327 PMC2938781

[15] Rothrock NE Amtmann D Cook KF. Development and validation of an interpretive guide for PROMIS scores. J Patient Rep Outcomes. (2020) 4:16. doi: 10.1186/s41687-020-0181-7, PMID: 32112189 PMC7048882

[16] Shouman K Vanichkachorn G Cheshire WP Suarez MD Shelly S Lamotte GJ. Autonomic dysfunction following COVID-19 infection: an early experience. Clin Auton Res. (2021) 31:385–94. doi: 10.1007/s10286-021-00803-8, PMID: 33860871 PMC8050227

[17] Carmona-Torre F Mínguez-Olaondo A López-Bravo A Tijero B Grozeva V Walcker M. Dysautonomia in COVID-19 patients: a narrative review on clinical course, diagnostic and therapeutic strategies. Front Neurol. (2022) 13:886609. doi: 10.3389/fneur.2022.886609 35720084 PMC9198643

[18] Mallick D Goyal L Chourasia P Zapata MR Yashi K Surani S. COVID-19 induced postural orthostatic tachycardia syndrome (POTS): a review. Cureus. (2023) 15:e36955. doi: 10.7759/cureus.36955 37009342 PMC10065129

[19] Blitshteyn S Whitelaw S. Postural orthostatic tachycardia syndrome (POTS) and other autonomic disorders after COVID-19 infection: a case series of 20 patients. Immunol Res. (2021) 69:205–11. doi: 10.1007/s12026-021-09185-5, PMID: 33786700 PMC8009458

[20] Goadsby PJ Holland PR Martins-Oliveira M Hoffmann J Schankin C Akerman S. Pathophysiology of migraine: a disorder of sensory processing. Physiol Rev. (2017) 97:553–622. doi: 10.1152/physrev.00034.2015, PMID: 28179394 PMC5539409

[21] Zhou Q Verne GN. New insights into visceral hypersensitivity —clinical implications in IBS. Nat Rev Gastroenterol Hepatol. (2011) 8:349–55. doi: 10.1038/nrgastro.2011.83, PMID: 21643039 PMC3437337

